# Numerical Analysis of the Bending Properties of Cathay Poplar Glulam

**DOI:** 10.3390/ma8105362

**Published:** 2015-10-19

**Authors:** Ying Gao, Yuxuan Wu, Xudong Zhu, Lei Zhu, Zhiming Yu, Yong Wu

**Affiliations:** 1 MOE Key Laboratory of Wooden Material Science and Application, Beijing Forestry University, Beijing 100083, China; wuyuxuanwood@gmail.com (Y.W.); zhuxudong5008@gmail.com (X.Z.); yuzhiming@bjfu.edu.cn (Z.Y.); 2 Beijing Key Laboratory of Wood Science and Engineering, Beijing Forestry University, Beijing 100083, China; 3 MOE Engineering Research Center of Forestry Biomass Materials and Bioenergy, Beijing Forestry University, Beijing 100083, China; 4 Beijing University of Civil Engineering and Architecture, Beijing 100044, China; zhulei@bucea.edu.cn; 5 Suzhou Grownhomes Co., Ltd., Suzhou 215105, China; wu.yong@crownhomes.cn

**Keywords:** structural glued-laminated timber (glulam), Chinese domestic Cathay poplar, mechanical tests, bending properties, checking calculation, formulae, finite element analysis

## Abstract

This paper presents the formulae and finite element analysis models for predicting the Modulus of Elastic (MOE) and Modulus of Rupture (MOR) of Cathay poplar finger-jointed glulam. The formula of the MOE predicts the MOE of Cathay poplar glulam glued with one-component polyurethane precisely. Three formulae are used to predict the MOR, and Equation (12) predicts the MOR of Cathay poplar glulam precisely. The finite element analysis simulation results of both the MOE and MOR are similar to the experimental results. The predicted results of the finite element analysis are shown to be more accurate than those of the formulae, because the finite element analysis considers the glue layers, but the formulae do not. Three types of typical failure modes due to bending were summarized. The bending properties of Cathay poplar glulam were compared to those of Douglas fir glulam. The results show that Cathay poplar glulam has a lower stiffness, but a marginally higher strength. One-component polyurethane adhesive is shown to be more effective than resorcinol formaldehyde resin adhesive for Cathay poplar glulam. This study shows that Cathay poplar has the potential to be a glulam material in China.

## 1. Introduction

Modern wooden structures have been used in many areas around the world. Glued-laminated timber (glulam) is a type of engineered wood product that can preserve the natural beauty of the source wood during construction [1,2]. Structural glulam has been widely used in Japan, North America and Europe [3], but Chinese domestic wood species are rarely used in the construction of structural glulam manufactured in China. It is thus important to study Chinese domestic wood species that could be applicable for use in structural glulam.

Cathay poplar (*Populus cathayana*) is one of the most abundant, fast-growing species in northern China and is cold resistant, fast growing, straight grained, medium and evenly textured and easy to manufacture. Cathay poplar is thus an excellent material for construction components [4,5].

Finite element analysis (FEA) is an efficient research method used in the wooden materials field. By establishing a finite element analysis model and post-processing, visual predictions can be obtained without specimen failures. In 1980, Foschi and Barrett put forward the Foschi-Barrett simulation model, which used the Monte Carlo method, to predict the Modulus of Rupture (MOR) of glulam. The Ala Tabiei [6] model simulated the nonlinearity of wood, and the Moses D.M. [7] model predicted the nonlinear deformation and brittle fracture of wood composite material. The Serrano [8] model is also a three-dimensional nonlinear FEA model, which predicted the properties of the glue layer, the size effect and tensile fracture properties when the wood was connected with bolts. Gao [9] proposed the FEA model of the deformation of timber-framed plywood panel dome structures, and the simulated results were found to be in good agreement with the results of the experiments under similar loading conditions. Frese [10,11,12] simulated the bending and tensile strengths of spruce and beech glulam. Qiu [13] simulated the crack propagation behavior of Chinese larch, and the numerical results correlated well with the experimental results. Based on the stiffness profile of each board and its location within the glulam, Kandler [14] established a numerical finite element model, which is able to predict the effective glulam stiffness with high accuracy.

The formula model is also commonly used to make numerical simulation of glulam. Kohler [15], Fink [16,17] and Jockwer [18,19,20] have published contributions about the formula model. Kohler presented a probabilistic approach for modelling the tensile strength and tensile stiffness properties of timber boards and finger joint connections of knot clusters based on the results of the experimental investigation. Fink presented a probabilistic approach for modelling the load-bearing capacity, the bending stiffness and the failure modes of glulam. In Jockwer’s research, the prediction of the load-carrying capacity of notched beams was developed based on experiments and theory, and the load at initial cracking of the reinforced notches tested can be predicted with the model. Besides, the impact of knots and grain deviations on the fracture perpendicular to the grain of timber is analyzed by means of numerical models.

The Modulus of Rupture (MOE) and MOR are both paramount properties of glulam. Four formulae were derived, and finite element analysis models were established to predict the MOE and MOR of Cathay poplar finger-jointed glulam. In addition, the bending properties of Cathay poplar glulam were examined and compared to those of Douglas fir glulam. A comparison of the test and simulation results was also performed.

## 2. Materials and Methods

### 2.1. Material Properties

Cathay poplar was obtained from the Beijing Longshun Wood Market, Beijing, China. Douglas fir was imported from North America (Can-Coast Development Corp, Vancouver, BC, Canada). The moisture content of the Cathay poplar and the Douglas fir ranged from 9% to 12%. The physical and mechanical properties of the Cathay poplar and Douglas fir lumber are shown in Table 1. Resorcinol formaldehyde resin adhesive (RF, Prefere 5837W, Dynea, Gaoyao, Guangdong, China) and one-component polyurethane adhesive (PU, HB S309, Purbond, Shanghai, China) were used to manufacture the glulam specimen. The resin content of RF and PU is 100% and 45%~75%, respectively; the curing time of RF and PU is 75 min and 60 min, respectively.

**Table 1:** Mean physical and mechanical properties of the lumber investigated in this study.

Species	Thickness (mm)	Lumbers Grades	Density (kg·m^−3^)	Moisture Content (%)	MOE (MPa)
Cathay poplar	20	I	536	10.7	12,798
		II	536	10.7	11,357
		III	536	10.7	9,900
	30	I	536	10.7	11,750
		II	536	10.7	11,297
		III	536	10.7	10,430
Douglas fir	20	II	498	9.2	12,500
	30	II	498	9.2	12,236

### 2.2. Grading of Sawn Lumber

Visual grading and the MOE test method were used in this study. A total of 52 pieces of Cathay poplar sawn lumbers with dimensions of 60 (width (W)) mm × 800 (length (L)) mm was tested, where half of the pieces were 20 mm thick and the other half were 30 mm thick. The lumber was graded based on GB/T (Recommended National Standards of China) 26899-2011 [21]. The concentrated knot’s diameter ratio (CKDR, the ratio of the diameters of all knots to the lumbers’ width in a 150 mm-long range) of Grades I, II and III lumber is not more than 20%, 30% and 40%, respectively.

**Figure 1:**
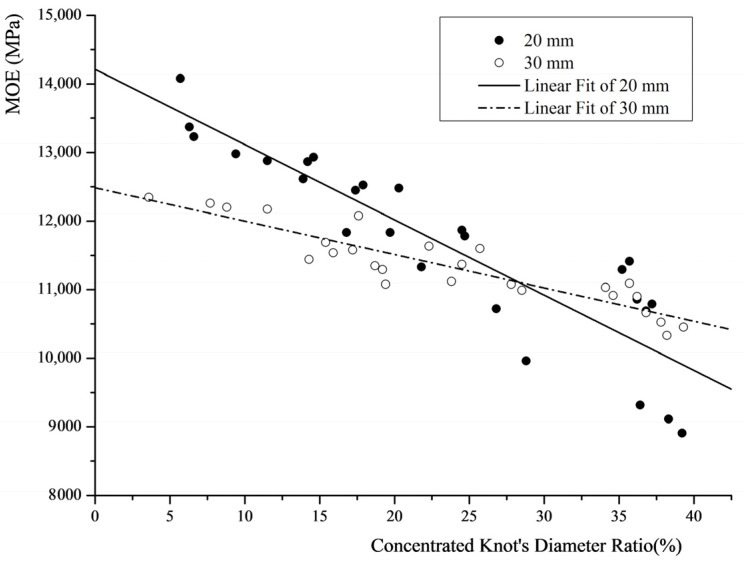
MOE of sawn lumber *versus* the concentrated knot’s diameter ratio.

Figure 1 shows that the MOE decreases as the ratio increases. When the ratio is less than 30%, the MOE of the 20 mm-thick lumber is higher than that of the 30 mm-thick (T) lumber. However, when the ratio is in the range of 30%~40%, the linear fit of the MOE of the 30 mm-thick lumber is higher than that of the 20 mm-thick lumber.

### 2.3. Manufacturer of Specimen

Glulam specimens were manufactured based on GB/T 26899-2011. All of the glulam specimens were 3000 (L) mm × 60 (W) mm × 120 (T) mm. The length of finger joints was 21.5 mm, and the width was 8.0 mm. The distribution of finger joints in glulam satisfies the construction requirement of glulam. The positions of finger joints were at least 30 cm away from each adjacent layer.

**Figure 2:**
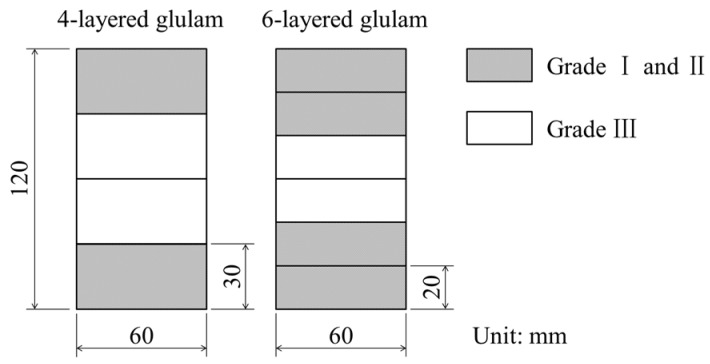
Cross-section of the glulam specimens.

The specimen parameters are shown in Table 2. To improve the bending strength of glulam [22], Grades I and II lumber were placed on the outermost layers of the 4-layer glulam and the outermost and outer layers of the 6-layer glulam. Grade III lumber was placed on the inner layers (Figure 2).

**Table 2:** Specimen parameters. RF, resorcinol formaldehyde; PU, polyurethane; W, width; T, thickness.

Species	Adhesive	Sectional Dimensions of Laminates (mm) *	Layers	Abbreviation	Sample Size
Cathay poplar	RF	60 (W) × 30 (T)	4	PR4	3
	RF	60 (W) × 20 (T)	6	PR6	3
	PU	60 (W) × 20 (T)	6	PO6	4
Douglas fir	PU	60 (W) × 30 (T)	4	DO4	5
	PU	60 (W) × 20 (T)	6	DO6	5

*: T represents the thickness of laminates.

### 2.4. Equipment and Experiment

**Figure 3:**
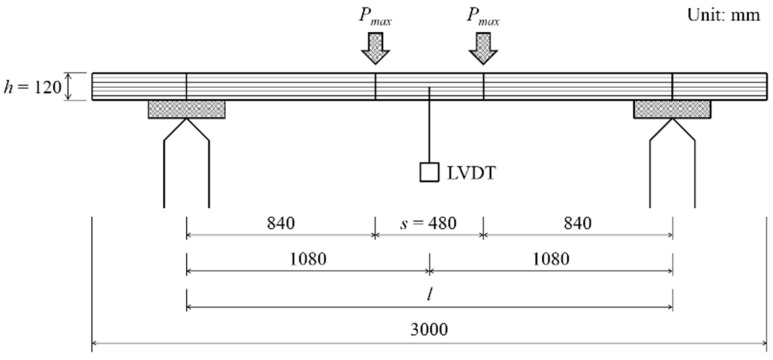
Bending test Method A (side view).

A four-point load static bending test (*i.e.*, bending test Method A) was performed based on GB/T 26899-2011 at a loading speed of 8 mm/min. An LVDT (Linear Variable Differential Transformer) sensor was set at the midspan of each specimen. (Figure 3). The MOE for bending and MOR are calculated by the following Equation:

(1) $$\text{MOE}=\frac{\text{Δ}P\left(l-s\right)\left(2l^{2}+2ls-s^{2}\right)}{8\text{Δ}ybh^{3}}$$

(2) $$\text{MOR}=\frac{3P_{max}\left(l-s\right)}{2bh^{2}}$$

where ΔP is the difference between the upper and lower loads at the proportional limit, *l* is the span of the specimen between supports, *s* is the span of the specimen between the loading points, Δ*y* is the corresponding midspan deflection of ΔP, *b* is the width of the specimen, *h* is the depth of specimen and *P_max_* is the maximum load.

## 3. Experimental Results and Discussion

### 3.1. Failure Modes

**Figure 4:**
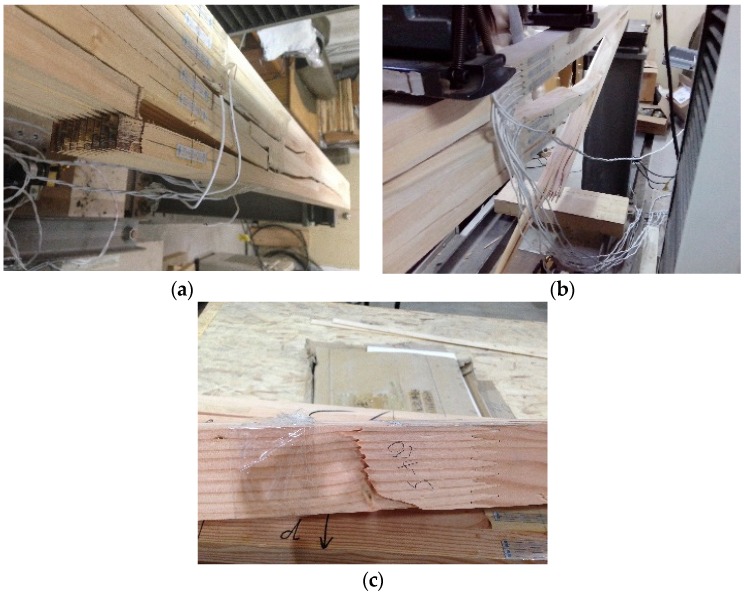
Typical failure modes of specimens. (**a**) Shear failure of finger joint (PR4 and PR6); (**b**) shear failure of finger joint and splitting of timber (PO6); (**c**) splitting of timber (DO4 and DO6).

Elastic deformation occurred in the specimens during the initial stage of loading. As the load increased, plastic deformation occurred in the specimens. At this stage, many small compression wrinkles appeared in the specimens. The bending rigidity of the specimen decreased marginally, and the deformations increased significantly. Finally, the bottom laminates reached their ultimate tensile stresses. The finger joints of the bottom laminate or defects in the timber failed first, which led to brittle failure of the specimen. The three typical failure modes observed are shown in Figure 4.

The failure modes were connected to the finger joint to some degree. Structural glulam requires a large number of finger joints, so studies of finger joint fatigue strength are very important [23]. There are quite a few research works aimed at the relationship between the bending strength of glulam and finger joint strength.

In terms of experimental research, Ohuchi [24,25] came to a conclusion based on bending tests of hinoki (*Chamaecyparis obtusa*) and sugi finger-jointed laminae. In these studies, to examine the optimum adhesive condition in the finger-joint part that influences the strength properties of the large-scale finger-jointed laminae, the block shear tests with block specimens that assumed the finger-joint part and glued under various adhesive conditions was performed. In conclusion, for glulam with good strength properties, the evaluation of finger-joint properties is important. In addition, Frese [26] tested the characteristic bending strength of beech glulam and characteristic finger joint bending strength. The conclusion was proposed that the bending strength of glulam depends on both board strength and finger joint strength.

In terms of the glulam strength model, Frese [11] put forward a glulam model, where its bending strength depends both on the board tensile strength and finger joint tensile strength. This model could determine the influence of the board and finger joint strength on glulam bending strength, which proved the relationship between the bending strength of glulam and finger joint strength. As a result, the poor characteristic bending strength values of the 38 test beams were mainly caused by too low requirements for boards and finger joints, which verified the model. In addition, Frese [10,27] proposed a predictive model for characteristic glulam bending strength based on tension tests of the boards and the finger joints. The experimental values of them were used as input data for the strength models to calculate the predictive values of the glulam. This model predicted the characteristic glulam bending strength precisely. Comparisons between the experimental data and the analytical results from the computer model show a good agreement. The predictive values for the characteristic glulam bending strength and the experimental ones differed not more than 6%. This indicated that the bending strength of glulam was determined by finger joint strength to a large extent.

Therefore, based on the above research, the following conclusions were proposed.

Shear failure of the finger joint led to the failures of PR4 and PR6. The bending strengths of PR4 and PR6 were indicative of the bonding strength of a finger joint with RF, but did not fully describe the mechanical properties of Cathay poplar.

Shear failure of the finger joint and splitting of timber caused the failure of PO6. The bending strength of PO6 was indicative of both the bonding strength of a finger joint with PU and the mechanical properties of Cathay poplar. Compared to the RF, the Cathay poplar glued with PU performs better.

Splitting of timber resulted in the failures of DO4 and DO6. The bending strengths of DO4 and DO6 were indicative of the mechanical properties of Douglas fir, but did not fully describe the bonding strength of a finger joint with PU.

### 3.2. Experimental Results

**Figure 5:**
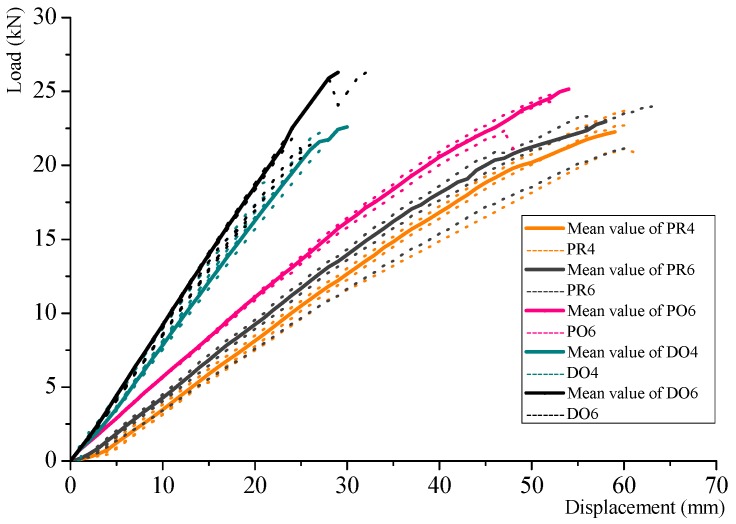
Load displacement curves of mean value of the specimens from each series and single results.

The load displacement curves of the mean value of the specimens from each series are shown in Figure 5 as solid lines, and the single results are shown as dotted lines. Compared to the Douglas fir glulam, the Cathay poplar glulam has a lower stiffness, but a slightly higher strength. The PU is shown to be more effective than the RF when used to glue the Cathay poplar glulam specimens. With the same thickness, the strength of the specimen increased when the number of layers increased.

**Figure 6:**
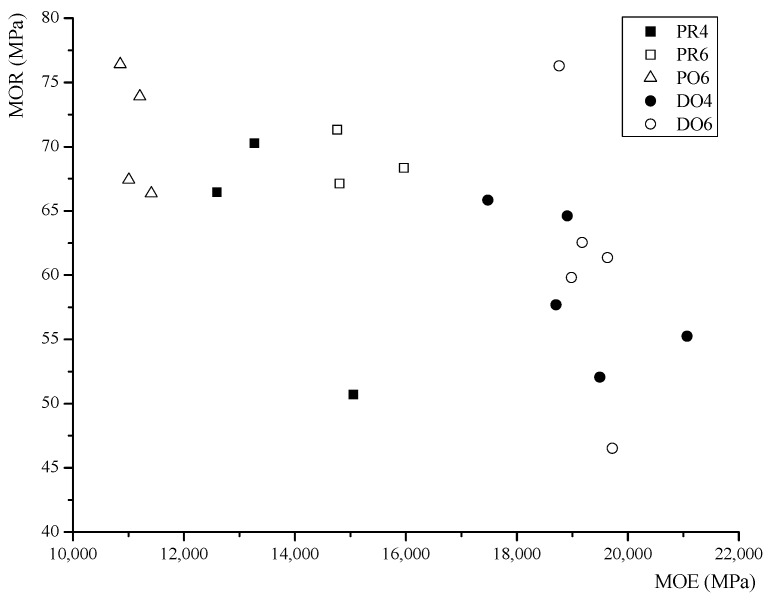
Scatter plot of MOR *versus* MOE for each series.

Figure 6 shows the relationship between the MOE and MOR. The mean values of each series are summarized in Table 3. Based on these results, the following conclusions are proposed.

**Table 3:** Experimental results of each series.

Specimen	Maximum Load *P*_max_ (kN)	Maximum Deflection (mm)	MOR (MPa)	MOE (MPa)	Bending Rigidity (MPa)	Ultimate Tensile Strain
PR4	23.24	59.67	68.37	8930	77.16 × 10^9^	0.0034
PR6	23.44	58.56	69.22	9243	79.86 × 10^9^	0.0047
PO6	24.33	49.75	71.04	11,119	96.07 × 10^9^	0.0053
DO4	22.33	27.86	65.22	18,195	157.20 × 10^9^	0.0032
DO6	23.77	28.96	69.41	18,971	163.91 × 10^9^	0.0038

Firstly, a comparison of the different wood species was performed. The MOR of PR6 was 2.4% higher than that of DO6, and the MOE of DO6 was 70.6% higher than that of PO6. In this study, the Cathay poplar glulam had a lower stiffness, but a marginally higher strength compared to those of the Douglas fir glulam. The ultimate tensile strain of PO6 was 0.0053, and the maximum compressive strain was 0.0063; these values were both higher than those of Douglas fir glulam.

Secondly, a comparison of the different adhesives was performed. Based on the experimental results, the MOR of PO6 was 2.6% higher than that of PR6, and the MOE of PR6 was 32.9% higher than that of PO6. For the Cathay poplar glulam, the PU is shown to be more effective than the RF.

Lastly, a comparison of the different numbers of layers was performed. The MOR of PR6 was 1.2% higher than that of PR4, and the MOR of DO6 was 6.4% higher than that of DO4. The MOE of PR6 was 14.3% higher than that of PR4, and the MOE of DO6 was 4.3% higher than that of DO4. With the same thickness, the bending strength increased as the number of layers increased.

### 3.3. Checking Calculation

The bending strength, the shear strength and the deflection of the glulam specimens were checked.

**Figure 7:**
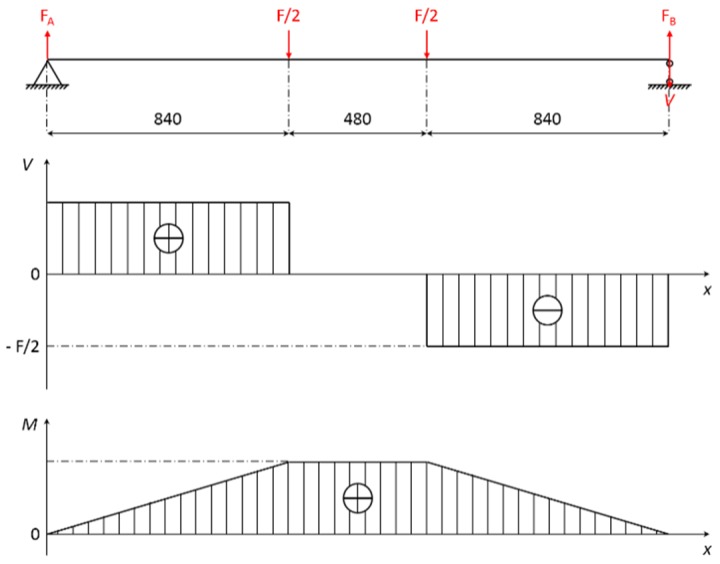
Shear diagram and bending moment diagram.

The load of a room was assumed to be carried by sample PO6. Based on the characteristic load combination in GB 50009-2012 [28], the design value of the load was 4000 N. According to the four-point load static bending test method, the design value of the maximum moment *M* and maximum shear strength *V* were 1.68 × 10^6^ N·mm and 2000 N based on the principle of the balance of force and moment, respectively. The shear diagram and bending moment diagram are shown as Figure 7.

The characteristic values (*i.e.*, 5%, the percentile of the sample) of the mechanical strength of the Cathay Poplar were calculated. Based on GB 50005-2003 [29], a national standard code for the design of timber structures issued by the Ministry of Housing and Urban-Rural Development of the People’s Republic of China, the Cathay poplar achieved a strength grade of TB (Tally of Broadleaf tree) 17. As shown in Figure 4.2.1-3 of GB 50005-2003, the wood species that achieved a strength grade of TB17 all have a design bending strength of 17 MPa and a design shear strength parallel to the grain of 2.4 MPa. Accordingly, the design values of the bending strength *f_m_* and the shear strength parallel to the grain *f_v_* were 17 and 2.4 MPa, respectively.

#### 3.3.1. Checking Calculation of the Bending Strength

The bending strength was checked by Equation (3). *S* is the section modulus, which was 1.44 × 10^5^ mm^3^, and M was 1.68 × 10^6^ N·mm.

(3) $$\frac{M}{S}\leq f_{m}$$

The value of checking calculation was 11.67 MPa, which was lower than the design value of bending strength *f_m_* (17 MPa). In conclusion, PO6 achieves the required bending strength in GB 50009-2012.

#### 3.3.2. Checking Calculation of the Horizontal Shearing Strength

The shearing strength was checked by Equation (4). *Q* is the first moment of area, which was 1.08 × 10^5^ mm^3^; *I* is the second moment of area (*i.e.*, moment of inertia), which was 8.64 × 10^6^ mm^4^; *b* is the width of the specimen, which was 60 mm; and V was 2000 N.

(4) $$\frac{VQ}{Ib}\leq f_{v}$$

The value of checking calculation was 0.42 MPa, which was lower than design value of horizontal shearing strength *f_m_* (2.4 MPa). In conclusion, PO6 achieves the required horizontal shearing strength in GB 50009-2012 [28].

#### 3.3.3. Checking Calculation of the Deflection

The deflection was checked by Equation (5). δ′ is the design value of the deflection, which was 8.64 mm, and δ is the calculated deflection, which was calculated by Equation (6).

(5) δ≤δ′

(6) $$\text{δ}=\frac{F\left(l-s\right)\left(2l^{2}-2ls-s^{2}\right)}{8Ebh^{3}}$$

The MOE of PO6 was 11,119 MPa, so the value of checking calculation was 8.14 mm, which was lower than the design value of deflection. In conclusion, PO6 satisfies the deflection requirements in GB 50009-2012 [28].

Therefore, the bending strength, shear strength and deflection of PO6 pass the checking calculations.

## 4. Derivations of Formulae

### 4.1. MOE of the Cathay Poplar Glulam

The MOE for bending of the Cathay poplar glulam was calculated based on that of sawn lumber using a formula that will now be described. The accuracy of the formula is also verified below.

Wang and Chang [30] showed that the strength of laminate was based on the bending stiffness of laminates (*e_j_I_j_* = the bending stiffness of the *j* laminate). The bending stiffness of a glulam specimen was the sum of the bending stiffness of each laminate layer under the non-glued layers, as shown in Equation (7) [31]:

(7) $$E_{b}I=\sum^{\text{​}}e_{j}I_{j}$$

where *E_b_* is the MOE of the glulam specimen; *I* is the section moment of inertia of the glulam specimen; and *e_j_I_j_* is the MOE multiplied by the section moment of inertia of the *j* laminate (*j* = 1, 2, 3…, 6), respectively.

Based on the parallel axis theorem from material mechanics [32], the section moment of inertia of laminates can be transformed into the section moment of inertia of the central plane of the glulam specimen as Equation (8):

(8) $$I_{j}=I_{j{}^{\circ}}+A_{j}{\left(d_{j}\right)}^{2}$$

where *I_j0_* is the section moment of inertia of the *j*-th laminate to the central plane of the glulam specimen; *A_j_* is the sectional area of the *j*-th laminate; and *d_j_* is the distance between the *j*-th laminate and the central plane of the glulam specimen.

Equation (8) was then substituted into Equation (7):

(9) $$E=\frac{1}{I}\overset{n}{\underset{j=1}{\sum }}E_{j}\left[I_{j{}^{\circ}}+A_{j}{\left(d_{j}\right)}^{2}\right]$$

The mean values of the theoretical results of Equation (9) are shown in Table 4. The error between the test and theoretical results of PO6 was 2.89%; thus, the formula predicts the MOE of PO6 accurately. However, the error of PR4 and PR6 is somewhat larger; this is probably because the shear failure of finger joint led to the failures of PR4 and PR6, and thus, the bending properties of PR4 and PR6 did not fully describe the mechanical properties of the Cathay poplar. Therefore, the calculated MOE of PO6 is more precise than that of PR4 and PR6 based on existing data. Moreover, Equation (9) would be validated with more data by further experiments to test its accuracy.

**Table 4:** MOE of the Cathay poplar glulam from the experimental tests and calculations.

Specimen	Experimental Results (MPa)	Theoretical Results (MPa)	Error (%)
PR4	8930	11,113	24.4
PR6	9243	11,441	23.8
PO6	11,119	11,441	2.9

### 4.2. MOR of the Cathay Poplar Glulam

The MOR of glulam is typically significantly better compared to that of sawn lumber due to the distribution of defects in the lumber [33]. The MOR of glulam is closely related to its material properties. To explore the relationship between the MOR of glulam and the tensile strength or the MOR of the lumber used, three formulae were derived.

#### 4.2.1. Equation Referenced EN 1194 [34]

Based on many experimental results, EN (European Norm) 1194 (Europe Standard Committee) has shown that the relationship between the MOR of the bending of glulam and the tensile strength of the lumber used is linear. A laminate coefficient λ is thus introduced and is expressed in the following two Equation:

(10) $$\begin{array}{c} f_{b,gl,k}=7.35+1.12f_{t,lam,k}\\ \text{λ}=1.12+7.35/f_{t,lam,k} \end{array}$$

where *f_b,gl,k_* is the characteristic MOR of glulam and *f_t,lam,k_* is the characteristic value of the tensile strength of the lumber.

The laminate coefficient λ is inversely proportional to the characteristic value of the tensile strength of the lumber *f_t,lam,k_*. For lumber that has higher tensile properties, its laminate coefficient is smaller than lumber that has lower tensile properties. Therefore, the bending properties of glulam made of worse lumber have greatly improved compared to glulam made of better lumber.

The correlation coefficient *r* = 0.945 and the two formulae are only suitable for glulam with a thickness of 600 mm. If the dimensions are different, the MOR should be multiplied by the volume factor *k* [35,36],

(11) $$\frac{F_{b}}{F_{b{}^{\circ}}}=C_{v}={\left(\frac{V_{{}^{\circ}}}{V}\right)}^{k}$$

where *F_b_* is the MOR of glulam with other dimensions, *F_b0_* is the MOR of glulam with standard dimensions, *V_0_* is the standard volume (130 mm × 300 mm × 6400 mm), *V* is the measured volume and *k* is the volume factor = 0.076 [35].

#### 4.2.2. Regression Analysis

**Figure 8:**
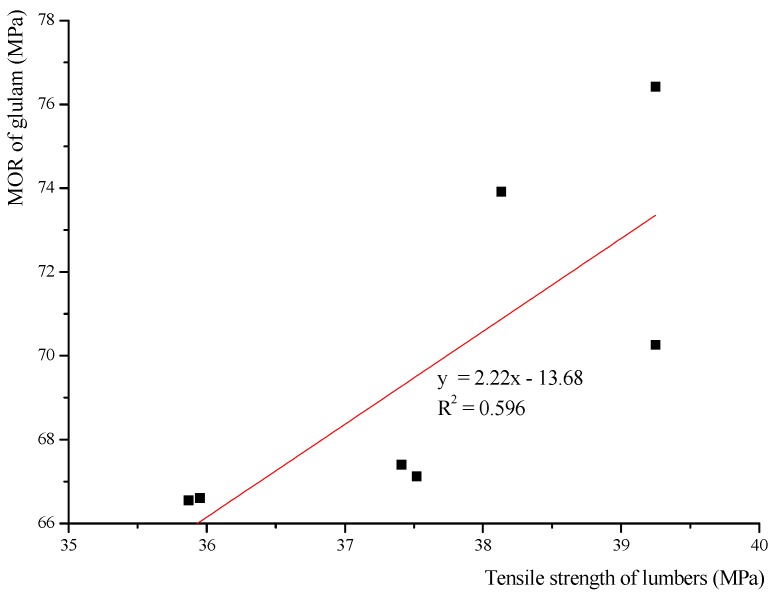
Linear regression analysis of the MOR of glulam.

Based on the bending and tensile results, regression analysis is used to describe the relationship between the MOR of glulam and the tensile strength of the lumber used. The tensile strengths of the lumber mean the mean experimental values of 20 mm-thick lumber and the mean experimental values of 30 mm-thick lumber. In this formula, the tensile strength of the lumber is an independent variable, and the MOR of glulam is a dependent variable. Based on the scatter diagram drawn by Origin (Figure 8), the relationship appears to be linear.

(12) $$f_{b,gl,k}=2.22\;f_{t,lam,k}-13.68\;\left(\text{R}2=0.596\right)$$

#### 4.2.3. Equation Based on the Parallel Axis Theorem

Equation (14) was derived to describe the relationship between the MOR of glulam and the MOR of the lumber used. For symmetrical glulam, the MOR is expressed by the following Equation:

(9) σ=Eε

where σ is the MOR of glulam, *E* is the MOE for the bending of glulam and ε is the elongation of a unit length.

Equation (8) in Section 4.1 was substituted into Equation (13) and Equation (14) to derive:

(14) $$\text{σ}=\text{ε}\times \frac{1}{I}\overset{n}{\underset{j=1}{\sum }}E_{j}\left[I_{j{}^{\circ}}+A_{j}{\left(d_{j}\right)}^{2}\right]$$

#### 4.2.4. Comparison between Test and Theoretical Results

The mean values of the theoretical results of Equations (10), (12) and (14) are shown in Table 5. In terms of existing data, Equation (12) predicts the MOR well, but the errors of Equations (10) and (14) are somewhat large. Moreover, these three formula would be validated with more data by further experiment to test their accuracy.

**Table 5:** MOR of Cathay poplar glulam from the test and theoretical results.

Specimen	Experimental Results (MPa)	Corrected Experimental Results (MPa)	Equation (10)		Equation (12)		Equation (14)	
			Theoretical Results (MPa)	Error (%)	Theoretical Results (MPa)	Error (%)	Theoretical Results (MPa)	Error (%)
PR4	68.37	56.79	47.55	−30.5	69.57	−1.8	52.61	−23.0
PR6	69.22	57.49	51.31	−25.9	70.24	1.5	76.53	10.6
PO6	71.04	59.01	51.31	−27.8	70.24	−1.2	76.53	7.7

The theoretical results are shown to be 28.03% higher than the corrected experimental results on average. This is probably because Equation (10) was derived based on the experimental results of European glulam, and their mechanical properties have many differences from that of Cathay poplar glulam.

Equation (12) was derived based on the experimental results, and the theoretical results are similar to the experimental results. Equation (12) can predict the MOR of Cathay poplar glulam.

Equation (14) shows that the error between the test and calculated values of PR4 is somewhat larger. This is because of the softening of a finger-joint bond. This deviation leads to 23.04% lower theoretical results compared to the experimental results.

## 5. Finite Element Analysis

**Figure 9:**
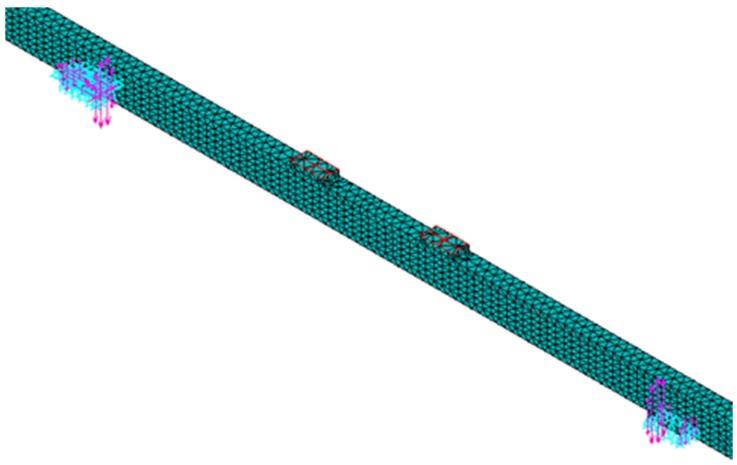
Meshed finite element model with loads and constraints.

The MOE and MOR of Cathay poplar glulam are calculated using the finite element program ANSYS, Version 14.5. Figure 9 shows the meshed finite element model with the loads and constraints. The “8 nodes solid 185” element was chosen, and the material properties were defined based on the experimental results of Cathay poplar glulam. Cathay poplar is orthotropic, and the glue layers (the MOE is 3000 MPa, and Poisson’s ratio μ is 0.37) are isotropic. Knots were not taken into consideration when the model was established. The compressive zone is assumed to be ideally elastoplastic, and the tensile zone is assumed to be ideally elastic. The calculation does not stop until the tensile stress in the center of an element lies in between a range ±0.5% of the tensile strength of the board section [10].

In addition, four elastic cushion blocks are set at two of the supports and two loading points to mitigate stress concentrations and to create a more accurate simulation. Based on He [37] and Tuohuti [38], the elastic cushion blocks are isotropic. The MOE is 1000 MPa, and Poisson’s ratio μ is 0.2.

The mean values of the simulation results of the MOE for bending and MOR of Cathay poplar glulam are shown in Table 6.

**Table 6:** MOE and MOR of Cathay poplar glulam from the test and simulation results.

Specimen	MOE			MOR		
	Experimental Results (MPa)	Simulation Results (MPa)	Error (%)	Experimental Results (MPa)	Simulation Results (MPa)	Error (%)
PR4	8930	9732	9.0	68.37	63.04	−5.3
PR6	9243	9952	7.7	69.22	65.49	−5.4
PO6	11,119	11,439	2.8	71.04	68.24	−3.9

For the MOE, the simulation results are all higher than the experimental results. The mean error of PR4 is 8.98%, while that of PR6 is 7.67%; the error of PO6 is lower (2.79%). Thus, the simulation of PO6 is more accurate than those of PR4 and PR6.

For the MOR, the simulation results are all lower than the experimental results. The mean error of PR4 is −5.33%, that of PR6 is −5.39% and that of PO6 is −3.89%. Thus, the simulation of PO6 is more accurate than those of PR4 and PR6.

Therefore, the simulations of the MOE and MOR of PO6 are better than those of PR4 and PR6.

**Figure 10:**
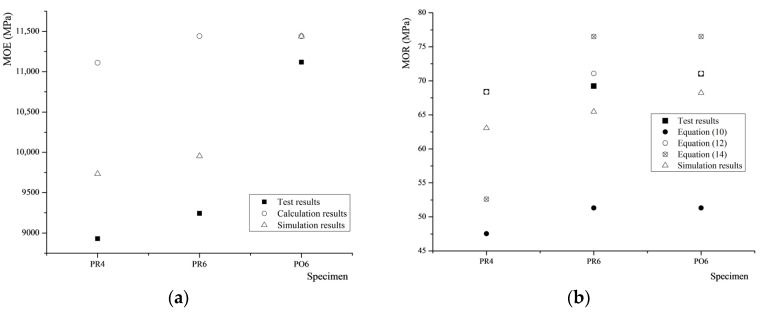
Experimental results of MOE (**a**) and MOR (**b**) compared to the calculation and simulation results.

Figure 10a compares the experimental results of the MOE for bending and MOR with the calculation and simulation results.

For the MOE, the calculation and simulation results of PO6 approach the experimental results, but the errors between the results of PR4 and PR6 are somewhat larger. Therefore, the formulae and simulation model are both suitable to predict the MOE of Cathay poplar glulam glued with PU.

For the MOR, the error between the test and simulation results is small, but the error between the test and theoretical results is somewhat larger. Therefore, the results of the finite element analysis are more accurate than those of the formulae. This is probably because the glue layers were considered in the simulation models, but the formulae were derived without considering the glue layers.

## 6. Conclusions

Based on the experiments, calculations and finite element analysis, the following conclusions can be drawn.

Cathay poplar glulam has a lower stiffness, but slightly higher strength compared to Douglas fir glulam in this experiment. The ultimate tensile strain of Cathay poplar glulam glued with polyurethane is 0.0053, and its maximum compressive strain is 0.0063, both of which are higher than those of Douglas fir glulam. This study indicates that Cathay poplar has the potential to be glulam material in China. In terms of the adhesive, the MOR of six-layer Cathay poplar glulam glued with one-component polyurethane adhesive is 2.6% higher than that of six-layer Cathay poplar glulam glued with resorcinol formaldehyde resin adhesive, and the MOE of the latter is 32.9% higher than that of the former.

A formula is derived to predict the MOE of Cathay poplar glulam based on the MOE of its laminates. The results indicate that the formula could predict the MOE of six-layer Cathay poplar glulam glued with one-component polyurethane adhesive. Three formulae are used to predict the MOR of Cathay poplar glulam based on the MOR and the tensile strength of its laminates. Equation (12) is suitable to predict the MOR of Cathay poplar glulam. The simulation results of both the MOE and MOR of Cathay poplar glulam are similar to the experimental results. The simulation of Cathay poplar glulam glued with one-component polyurethane is shown to be more accurate than those of Cathay poplar glulam glued with resorcinol formaldehyde resin adhesive. The error of the MOE is shown to be 2.8%, and that of the MOR is shown to be −3.9%.
